# Severe Rhabdomyolysis Associated with Simvastatin and Role of Ciprofloxacin and Amlodipine Coadministration

**DOI:** 10.1155/2015/761393

**Published:** 2015-03-26

**Authors:** Nicolas De Schryver, Xavier Wittebole, Peter Van den Bergh, Vincent Haufroid, Eric Goffin, Philippe Hantson

**Affiliations:** ^1^Department of Intensive Care, Cliniques Universitaires Saint-Luc, Université Catholique de Louvain, 1200 Brussels, Belgium; ^2^Centre de Référence Neuromusculaire, Cliniques Universitaires Saint-Luc, Université Catholique de Louvain, 1200 Brussels, Belgium; ^3^Louvain Centre for Toxicology and Applied Pharmacology, Cliniques Universitaires Saint-Luc, Université Catholique de Louvain, 1200 Brussels, Belgium; ^4^Department of Nephrology, Cliniques Universitaires Saint-Luc, Université Catholique de Louvain, 1200 Brussels, Belgium

## Abstract

Simvastatin is among the most commonly used prescription medications for cholesterol reduction and the most common statin-related adverse drug reaction is skeletal muscle toxicity. Multiple factors have been shown to influence simvastatin-induced myopathy. In addition to age, gender, ethnicity, genetic predisposition, and dose, drug-drug interactions play a major role. This is particularly true for drugs that are extensively metabolized by cytochrome P450 (CYP)3A4. We describe a particularly severe case of rhabdomyolysis after the introduction of ciprofloxacin, a weak CYP3A4 inhibitor, in a patient who previously tolerated the simvastatin-amlodipine combination.

## 1. Introduction

Extremely severe rhabdomyolysis with statin therapy remains rare, in comparison with myalgias or mild elevation of muscular enzymes. Among precipitating factors, drug-drug interactions are playing an important role and should be investigated in depth. We are reporting a case of impressive rhabdomyolysis occurring soon after the introduction of antimicrobial therapy in patient who acquired peritoneal dialysis-related peritonitis.

## 2. Case Report

A 41-year-old African woman presented to the hospital for diffuse myalgia and intense weakness developing over the last 3 days. Her previous medical history included systemic lupus erythematosus (SLE) complicated by severe glomerulonephritis. After progression of renal failure, she required peritoneal dialysis (PD) for the last 3 years. She was on automated PD with 3 exchanges of 1,800 mL during the night and fill volume of 1,500 mL with icodextrin during daytime. Current medications included furosemide 125 mg b.i.d., amlodipine 10 mg o.d., bisoprolol 10 mg o.d., irbesartan 300 mg o.d., mycophenolate mofetil 500 mg o.d., hydroxychloroquine 200 mg b.i.d., calcium carbonate 1 g b.i.d., colecalciferol 625 *μ*g weekly, sevelamer 800 mg b.i.d., calcitriol 0.25 *μ*g three times a week, and simvastatin 20 mg o.d. Adherence to treatment was good and no modification in type or doses of any of these medications was done since the last 12 months. The patient had a residual creatinine clearance of 5 mL/min. Nine days before admission, she was diagnosed with a peritoneal dialysis-related peritonitis (PDRP) based on the cloudy aspect of the peritoneal dialysis effluent (PDE) with 1,300 cells/*μ*L and 47% neutrophils. She was at this time poorly symptomatic with no fever or abdominal discomfort and was given empiric antimicrobial therapy with vancomycin 2,000 mg/3d, ciprofloxacin 500 mg b.i.d., and a single dose of gentamicin 80 mg according to our protocol [1]. Four days later, culture of the PDE grew for* Corynebacterium striatum* and ciprofloxacin was withdrawn. The next day, she started to complain of diffuse severe muscle pain with intense weakness and the PDE color appeared reddish. The patient became also progressively anuric. On admission, laboratory investigations showed an impressive rhabdomyolysis with creatine kinase (CK) 540,000 IU/L (*N* < 200), lactate dehydrogenase (LDH) 19,200 IU/L (*N* < 248), and aspartate aminotransferase 1,700 IU/L (*N* < 50). Urine output before the incidence of rhabdomyolysis was 1,500 mL/day. Serum creatinine value peaked at 12.4 mg/dL (usual value was 9 mg/dL). Severe electrolyte disorders were observed with potassium 6.3 mmol/L, phosphorus 12.8 mg/dL, and ionized calcium 2.9 mg/dL. The patient denied any recent ingestion of a substance or drug other than the prescribed antimicrobial therapy and she reported no recent strenuous exercise. Urine toxicological analysis was negative for opioids and cocaine. No biological sign was consistent with a progression of SLE (anti-DNA antibodies 3.2 U/mL (*N* < 8), C3 and C4 complement components, respectively, 82 mg/dL (*N*: 85–193) and 38 mg/dL (*N*: 10–40)). Thyroid function tests were normal. Blood cultures remained sterile and Epstein-Barr virus, cytomegalovirus, human immunodeficiency virus, and influenza A and B serologies were negative.

She was admitted to the intensive care unit (ICU) for the correction of electrolyte disorders and fluid balance. Serum CK peaked at 816,000 IU/L on day 2 and then progressively decreased. Peritoneal dialysis therapy was maintained (3 exchanges/day) and PDE progressively cleared. Peritonitis also progressively resolved. Renal function recovered partially with spontaneous reapparition of dark tea-colored urine output (600 mL/d). A right vastus lateralis needle muscle biopsy was performed. The biopsy was of small size and was normal except for increased size and amount of lipid droplets. Serum carnitine and acylcarnitine levels were normal. Lymphocyte carnitine palmitoyl-CoA transferase 2 activity was increased.

Genetic analysis was performed to identify potential gene variants responsible for altered absorption, distribution, metabolism, and excretion of statins and/or for higher susceptibility to myopathy. The patient was cytochrome P-450 (CYP) 3A4*∗1/∗1* (absence of reduced activity* ∗22* allele), CYP3A5*∗1/∗1* (absence of inactive* ∗3* allele rending the patient CYP3A5 expressor), influx transporter gene* SLCO1B1∗1b/∗1b* (absence of reduced activity* ∗5* allele considered as the main genetic risk factor for developing myopathy after simvastatin intake), and efflux transporter gene ABCB1 heterozygote 3435CT. Electrolyte disorders were corrected and the patient left the ICU on day 3. At 3-month follow-up, serum creatinine returned to previous values, while the patient was still on PD and had a complete functional recovery.

## 3. Discussion

This patient developed a severe rhabdomyolysis soon after the introduction of an antimicrobial therapy for a PDRP. The pathogen that was identified, namely,* Corynebacterium striatum*, is not the most common one but was still found in 5% of culture positive PDRP [2]. It is usually transmitted from the skin following errors of manipulation by the patient. The paucity of symptoms and the poor neutrophilic response are frequent and consistent with the indolent pattern of this microorganism.

Among the main etiologies of rhabdomyolysis (traumatism, exertion, muscle hypoxia, genetic defects, infection, hypo- or hyperthermia, and metabolic and electrolyte disorders), drugs and toxins are playing a significant role [3, 4]. Among the latter, statins, fibrates, alcohol, heroin, and cocaine are the commonest agents.

Statins are widely prescribed in developed countries. Although generally safe, they can induce muscle disorders ranging from myalgias without any biologic abnormality to life-threatening rhabdomyolysis. Statin-related rhabdomyolysis (SRR), usually defined as CK levels exceeding 10 times the upper limit of normal range, is reported to be very infrequent (in comparison with myopathy or myalgias), with an incidence of 0.01% for five-year therapy [5], that is, 0.04–0.12 cases per million prescriptions [6]. A recent review of literature data (1990–2013) identified 112 cases classified as rhabdomyolysis, and simvastatin was frequently cited (55/112) [7]. In about half of the cases of SRR, rhabdomyolysis could be precipitated by an interaction between statin and a new prescribed drug that is believed to interfere with statin metabolism [6, 7].

Statins metabolism varies from one type to another. After passive absorption by the intestinal cells, simvastatin is transported from the portal blood to the hepatocytes by an influx pump, the organic anion-transporting polypeptide 1B1 (OATP1B1). Simvastatin is an inactive lactone that is then predominantly (≥80%) metabolized by CYP3A4 and 3A5 with a little contribution of CYP2A8 (≤20%) into *β*,*δ*-dihydroxy acid, its active metabolite [8]. The metabolism is almost complete after hepatic first pass. Drugs inhibiting CYP3A4 such as azole antifungals, HIV protease inhibitors, macrolides, cyclosporine, verapamil, diltiazem, amiodarone, and grapefruit juice may increase serum simvastatin level and its toxicity. Sensitivity to those interactions varies from one patient to another due to heterogeneous CYP3A4 activity among individuals [6]. Ciprofloxacin is a known weak inhibitor of CYP3A4 and may thus inhibit simvastatin metabolism, increasing its myotoxicity. Amlodipine has also been shown to competitively inhibit the metabolic activity of CYP3A4/5. A pharmacokinetic interaction model revealed that coadministration of simvastatin with 10 mg amlodipine could significantly increase simvastatin bioavailability and decrease simvastatin clearance, but also that these changes could be minimized by using a simvastatin dose lower than 24 mg [9, 10].

While an interaction between statins and other drugs on the CYP3A4 metabolic pathway is well known, the implication of other mechanisms has been suggested. Among these, glycoprotein P (P-gp) inhibition could play a role. P-gp is an active tissue-specific drug transporter belonging to the ATP-binding cassette superfamily. Acting as an efflux pump, it avoids cellular uptake of several drugs and toxins, preventing notably their absorption through the gastrointestinal tract. As simvastatin is not only an inhibitor but also a substrate of the P-gp, inhibition of P-gp by other drugs may increase serum levels of simvastatin when coadministered [11]. If P-gp interaction between ciprofloxacin and simvastatin has been suggested in a similar case report, there is currently still controversy to whether ciprofloxacin could be a substrate of P-gp [12]. Additionally, amlodipine is devoid of P-gp inhibiting properties [10].

The multiple drug protein 2 (MRP2) is another membrane transport protein from the ABC superfamily excreting toxins into bile that could play a role in drug-drug interactions as simvastatin is also a substrate of this transporter [13].

Several genetic factors may predispose to statin-related toxicity [14]. If gene variants coding for CYP3A4/5, the mitochondrial enzyme GATM, the influx transporter OATP1B1 (encoded by* SLCO1B1*), and the efflux transporters P-gp (encoded by ABCB1) have a theoretical potential for affecting absorption, distribution, metabolism, and excretion of statins, only the* ∗5* allele of* SLCO1B1* has been strongly identified to be clinically associated with statin induced myopathy, especially for simvastatin [14, 15]. The* SLCO1B1∗5* allele disrupts the localization of the OATPB1 influx transporter reducing the hepatic uptake of statins and increasing their plasma level and so their toxicity. Our patient was not found to be carrier of this allele. Her 3435CT heterozygote status for ABCB1 could not be theoretically associated with a decreased P-gp activity.

To date, only two other cases of rhabdomyolysis after drug interaction between simvastatin and ciprofloxacin have been reported, but none with a simvastatin-amlodipine interaction [16, 17].

Several other risk factors for SRR could also have influenced the severity of the rhabdomyolysis in our patient. First, CK levels are known to be higher in black people [18]. Secondly, SLE may be associated with a certain degree of latent myositis, even if no sign of progression of SLE was found in our patient. Third, the patient had terminal renal failure requiring PD. However, if statin-induced myopathy has been reported to be slightly increased among patients with renal failure [15], simvastatin does not seem to increase muscle disorders in patients with chronic kidney disease, even requiring peritoneal dialysis [19, 20].

In conclusion, this case illustrates the potentially dangerous interactions of some statins (simvastatin) with drugs that are also metabolized by the CYP3A4/5 pathway, even in the absence of genetic predisposing factors. While the US Food and Drug Administration (FDA) has advised that the daily dose of simvastatin should not exceed 20 mg in patients taking amlodipine concomitantly, the introduction of a new drug, even considered as a weak CYP3A4 inhibitor like ciprofloxacin, could increase the risk of myotoxicity [21].
